# An ontological modeling approach for abnormal states and its application in the medical domain

**DOI:** 10.1186/2041-1480-5-23

**Published:** 2014-05-21

**Authors:** Yuki Yamagata, Kouji Kozaki, Takeshi Imai, Kazuhiko Ohe, Riichiro Mizoguchi

**Affiliations:** 1 ISIR, Osaka University, 8-1 Mihogaoka, Ibaraki, Osaka, Japan; 2 Department of Medical Informatics, Graduate School of Medicine, The University of Tokyo, 7-3-1 Hongo, Bunkyo-ku, Tokyo, Japan; 3 Research Center for Service Science, School of Knowledge Science, Japan Advanced Institute of Science and Technology, 1-1 Asahidai, Nomi, Ishikawa, Japan

**Keywords:** Ontology, Abnormal state, Disease, Property, Attribute, Interoperability

## Abstract

**Background:**

Recently, exchanging data and information has become a significant challenge in medicine. Such data include abnormal states. Establishing a unified representation framework of abnormal states can be a difficult task because of the diverse and heterogeneous nature of these states. Furthermore, in the definition of diseases found in several textbooks or dictionaries, abnormal states are not directly associated with the corresponding quantitative values of clinical test data, making the processing of such data by computers difficult.

**Results:**

We focused on abnormal states in the definition of diseases and proposed a unified form to describe an abnormal state as a “property,” which can be decomposed into an “attribute” and a “value” in a qualitative representation. We have developed a three-layer ontological model of abnormal states from the generic to disease-specific level. By developing an *is-a* hierarchy and combining causal chains of diseases, 21,000 abnormal states from 6000 diseases have been captured as generic causal relations and commonalities have been found among diseases across 13 medical departments.

**Conclusions:**

Our results showed that our representation framework promotes interoperability and flexibility of the quantitative raw data, qualitative information, and generic/conceptual knowledge of abnormal states. In addition, the results showed that our ontological model have found commonalities in abnormal states among diseases across 13 medical departments.

## Background

With the development of newer technologies, data and information exchange have been required for several applications such as electronic health records (EHR) in medicine. Such data and information include abnormal states. However, abnormal states are difficult to share because of their heterogeneity, caused by the variety of grain sizes, from the level of cells, tissue, and organs to that of the entire human body. This results in diverse representations with little uniformity. BFO [1,2] and DOLCE [3] have contributed to the formalization of the quality description of entities. BFO provides E (Entity), P (Property) (e.g., <Eye (E), red (P)>) and DOLCE provides E (Entity), A (Attribute), V (Value) triple (e.g., <esophagus (E), length (A), short (V)>). However, we found that there are more complicated forms of quality representations in medicine. For example, “hypertension” is a compound concept, which has three elements: blood, pressure, and high joined to form one concept/word. Another example is “hyperglycemia,” composed of four concepts: blood, glucose, concentration, and high. Furthermore, in the case of “intestinal polyposis,” it is unclear whether “intestine” or “polyp” should be considered as the entity.

This motivated us to establish a common framework for the representation of abnormal states supported by sound theories. In this study, we investigate the representation of abnormal states from the content-oriented view, which focuses on how to capture the content to be represented, on the basis YAMATO [4].

YAMATO has been built to target both high utility and philosophical soundness while maintaining compatibility with BFO and DOLCE. In brief, YAMATO has the following characteristics:

a) Quality-related concepts (dependent continuant entities) are divided into “Property,” “Generic quality,” and “Quality value.” “Quality” in BFO is identical to “Property” in YAMATO.

b) “Quality value” is classified in a manner identical to the classification in scales of measurement.

c) The context dependency of “Ordinal value” is represented by using the theory of “Role”.

d) Multiple kinds of informational entities are symbolically represented.

For the quality description, representations with both EAV and EP formalisms are defined. Furthermore, PATO2YAMATO aims to integrate phenotype >descriptions that exist in different structured comparison contexts [5]. It allows (1) the classification of quality values, in which scales of measurements are properly represented; (2) strict modeling of the context dependency of ordinal values; and (3) clear distinction between “true values” and “measured data.” It provides the mapping of ontology terms of PATO [6] to YAMATO’s framework and enables the interoperability of the quality framework between different top-level ontologies such as BFO and DOLCE. For example, in the YAMATO framework, PATO:0000582 (increased weight) is defined as a Property that is a combination of Generic quality (Attribute), weight, and a context-dependent Quality value (Attribute Value), heavy. The context-independent value is defined as a class “Weight quality value.”

Another issue is that in several medical textbooks or medical dictionaries, abnormal states in the definitions of diseases have not been directly associated with the corresponding quantitative values of clinical test data (e.g., “ischemia” in ischemic heart disease or “muscular weakness” in muscular dystrophy), which makes their processing by a computer difficult.

Furthermore, clinicians often deal with abnormal states specific to each disease only in a particular medical division, which makes it difficult to spread awareness regarding the common nature of abnormal states. To address these issues, we have been developing abnormality ontology for the systematization of knowledge regarding abnormal states, using ontological engineering, which represents a unified framework [7]. We focus on abnormal states in the definitions of diseases, which should be referred to in several applications. In addition, we discuss the representation of the various abnormal states on the basis of ontological theories in a consistent manner.

Our claim in this study is not isolated to adopting one of the representational forms used in the existing resources. The aim of our work is to formalize and organize different representations used in clinical medicine on the basis of ontological theories, and to realize the interoperability between them. It's not a simple matter of the use of existing resources such as PATO, LOINC [8], and others. Unified theoretical considerations make the various representation forms interoperable, which enables the establishment of a consistent and computer understandable model for abnormal states that are used in the definitions of diseases and medical data.

In this study, we first define abnormal states and explain our representation model. Then, we introduce our ontology of abnormal states and demonstrate an application of our work. We have constructed a disease ontology and captured a disease as one or more causal chains of the abnormal states in the human body [9]. Till date, clinicians have described the causal chains of approximately 21,000 abnormal states for approximately 6,000 diseases across 13 medical departments. Thus, we believe that the use of our ontology will contribute to various clinical applications.

## Results

### Definition of abnormal states

In the human body, abnormal states are highly diverse and involve various grain sizes, from the level of cells, tissue, and organs to that of the whole organism. Therefore, to systematize the knowledge about abnormal states, it is important to clarify the essential characteristics of the abnormal states, and to conceptualize them in a consistent manner.

In this section, we focus on the abnormal states that appear in the definition of diseases rather than in reality. A “state” is modeled as a time-indexed property^a^ that is associated with an entity, and has the value of an attribute that changes with time [10]. For example, imagine the state of hunger. It is represented by “being hungry” or not at some time point in time. We define “Property” as a characteristic that is inherent in an entity, having an attribute along with its value, such as “being red”: <color, red>. Properties are distinct from attribute values; for example, the Property “hypertension” is differentiated from an Attribute Value such as “high” as in “blood pressure is high.” An Attribute Value has three subclasses: categorical value (e.g., viviparous/oviparous), quantitative value (e.g., 160 mmHg), and qualitative value (e.g., high/low, large/small, much/few). On one hand, the Attribute Value “high” can be used for several attributes such as temperature, density, and velocity. On the other hand, the Property “hypertension” cannot be used for the values of the abovementioned attributes and it has a set of attributes and values like < pressure, high >.

In several textbooks and dictionaries, diseases have been defined in terms of abnormal states. For example, the definition of diabetes is “Diabetes mellitus is characterized by chronic hyperglycemia with disturbances of…” [11]. Another disease myocardial ischemia is presented as “The term acute myocardial infarction should be used when there is evidence of myocardial necrosis with acute myocardial ischemia” [12]. Therefore, we can say that a disease can be defined in terms of an assertion about the patient “being in an abnormal state or not”.

In the medical domain, various types of representations for abnormal states are used, and we conceptualize these representations into three categories:

(1) Quantitative representation (e.g., blood pressure is 180 mmHg, blood glucose concentration is 135 mg/dL).

(2) Qualitative representation (e.g., blood pressure is high, blood glucose concentration is high).

(3) Property representation (e.g., hypertension, hyperglycemia).

Because the upper ontology YAMATO [4] has been carefully designed to cover the property, quality, and quantity ontologies, it supports our work on abnormal states.

A quantitative representation is important for diagnosis because a concrete value should be identified by clinical examination for each patient. However, in the definition of a disease, a property such as “being hypertensive” or “being hyperglycemic” is essential instead of quantitative data. Thus, as our basic policy, we first capture the abnormal states as properties, represented by a tuple like < Property (P), Property Value (Vp)>. The Property Value takes a binary value, i.e., <true/false>. For example, if the state “stenosis” exists, it is described as < stenosis, true>. In addition, when necessary, a Degree Value (Vd) can be used for describing the degree of the Property Value, such as < stenosis, severe >.

Some readers may think that a property represented in the above manner is extremely conceptual to be of practical use because of the lack of a representation, which would give a more concrete meaning to data. Therefore, we specify a property by decomposing it into a tuple: <Attribute (A), Attribute Value (V)>. The Attribute Value can be either a Qualitative Value (Vql) or a Quantitative Value (Vqt). For example, in a case of a qualitative representation, stenosis (P) is decomposed into < cross sectional area (A), small (Vql)>, and in another case of a quantitative representation, stenosis (P) is described as a concrete value, e.g., <cross sectional area (A), 5 mm^2^ (Vql)>. This approach contributes to promoting consistency in representation, as well as the interoperability between the quantitative raw data and the generic/conceptual knowledge regarding abnormal states (see after the section Interoperability between properties and attributes).

In clinical medicine, decomposition of some properties cannot be achieved, because the precise mechanisms in the human body have not yet been completely uncovered. For example, in the case of nausea, property representation could be nondecomposable. Whether such abnormal states represented in terms of properties defined above can be decomposed into a known attribute and its value will depend on advances in medicine.

### Representation of abnormal states

#### Basic representation

In this section, we introduce our representation model for clinical abnormal states and show that we can appropriately represent them in a consistent manner.

Because an attribute cannot exist by itself but always exists in association with an independent object, we need to identify the object (hereinafter referred to as “target object”). For example, in the case of “gastrectasia,” the target object of its attribute “volume” is the stomach. Accordingly, we introduce the “Object” to represent the target object of the attribute and decompose the property into a triple: <Object (O), Attribute (A), Attribute Value (V)>. This is our basic representation model for abnormalities. For example, “gastrectasia” is decomposed into < stomach, volume, large > ^b^ (Table 1(a), row 1).

**Table 1:** Representations of abnormal states

**(a) Representation**	**Abnormal states Property (P)**	**Property Value (Vp)**	**Attribute (A)**	**Attribute Value (V)**	**Object (O)**	**Sub-Object (SO)**	
Basic representation	Gasrtectasia (gastric dilation)	True	Volume	Large	Stomach		
	Nausea	True			Patient		
Extended representation	Hyperglycemia	True	Concentration	High	Blood	Glucose	
	Gastric polyposis	True	Number	Many	Stomach	Polyp	
**(b) Variant of Ratio**	**Abnormal states Property (P)**	**Property Value (Vp)**	**Attribute (A)**	**Attribute Value (V)**	**Object (O)**	**Sub-Object (SO)**	**Ratio**
m/n (no unit)	High m ratio	True	Ratio	High	The whole	Focused	m/n
Example	Hyperglycemia	True	Concentration	High	blood	glucose	Glucose/Blood
m/n (focused on m of same object)	High m ratio	True	Ratio	High	Object	m	m/n
Example	High Albumin ratio	True	Concentration	High	Urine	Albumin	Albumin/Creatinine
m/n (focused on the ratio of same object)	High m/n ratio	True	Ratio	High	Object		m/n
Example	Increased A/G ratio	True	Ratio	High	Blood	A/G	Albumin/Globulin

(a): Basic and extended representations (b): Representations of ratios.

### Extended representation

We recognize that some properties may be difficult to decompose into the basic triple representation, such as a ratio and what we call a meta-attribute, discussed below. Accordingly, we introduce a “Sub-object” (SO) to represent a focused object (see next paragraph) as an extended representation, so that a property can be decomposed into a quadruple: <Object (O), Sub-Object (SO), Attribute (A), Attribute Value (V) >.

In the case of a ratio, in addition to identifying the target object with the ratio, it should represent for what will be focused on (“focused object”). Therefore, we introduce a Sub-Object (SO) to represent a focused object. For example, the representation of “hyperglycemia” is a quadruple, <blood (O), glucose (SO), concentration (A), high (V)>, where the Object is blood and the Sub-Object is glucose [Table 1(a), row 3].

There appear to be different kinds of ratios depending on what is focused on. As a result, the Object and Sub-Object vary according to the type of ratio. Our representation model can represent all of them, as shown in Table 1(b). A detailed discussion can be found in a report by Yamagata Y et al. [13].

Furthermore, we show the representation of a meta-attribute. In the case of the property “gastric polyposis,” although color and size are attributes of polyps, “many polyps” is not an attribute of “polyps” because it is not inherent in each polyp. Following the meta-attribute approach in YAMATO, where, in the case of “the road is curvy,” “number of curves” is identified as a meta-attribute of curves, and the road, which has many curves, can be represented in terms of it (the number curves). Accordingly, we regard “the number of polyps” as a meta-attribute of polyps and the stomach can be described in the same manner as a road. By introducing “Sub-object,” the property “gastric polyposis” can be decomposed into a quadruple < stomach (O), polyps (SO), number (A), many (V)>, where the stomach is identified as the Object, and the polyps as the Sub-object [Table 1(a), row 4].

### Interoperability between properties and attributes

Our claim in this clause is the interoperability between the abnormal states and data. Considering the interoperability between the abnormal states and clinical test data, the OP form itself may not be compatible with the observational data.

A large amount of clinical test data is stored in hospitals. To ensure the cross-compatibility between those data and the abnormal states (described in EHR), the unified form should be required for computer processing, so that an exchange mechanism between the OP and OAV is indispensable.

The OAV form can deal with both the quantitative and qualitative representation of values. Most clinical test data are quantitative, e.g., the arterial cross sectional area of 24 mm^2^ and can be represented by OAVqt as < artery (O), cross sectional area (A), 24 mm^2^ (Vqt) > in our model. Notably, the quantitative value can be converted to a qualitative value such as “small (Vql),” with the threshold given by each hospital.

In the case of an abnormal state “arterial stenosis,” we can guarantee the interoperability between the quantitative data and abnormal state by decomposing “stenosis” into “cross sectional area is small”, which is represented as < artery (O), cross sectional area (A), small (Vql) >.

Another example is the quantitative data “blood glucose concentration level is 260 mg/dL”. It is represented by < blood (O), glucose (SO), concentration (A), 260 mg/dL (Vqt)>, and the interoperability between the quantitative test data and the abnormal states “hyperglycemia” used in the definition of diabetes is realized via qualitative representation such as < blood (O), glucose (SO), concentration (A), high (Vql) > in the extended OSoAV form.

Furthermore, another issue is the requirement of the degree value of the property. Clinicians usually need to transform test data into abnormal states in the case report. Imagine a case where a highly elevated value is observed in the clinical test. A simple OP < O, P, true > does not satisfactorily capture such data, and thus the degree of abnormal states may be needed. Therefore, we introduce the Degree Value (Vd) like “severe”. Therefore, we can describe such data in terms of the degree value in the OPVd form < blood (O), hyperglycemia, severe (Vd) > in a triplet like in the OAV form.

We recommend that the degree value should only have minimum variations such as mild/moderate/severe for representation because numerous degree values would lead to dispersion and destruction of the unified representation. Taking the interoperability into account, it would be considered preferable to decide how to set a threshold for determining the degree value of “severe”. However, because such concrete threshold values tend to change with time, such threshold values are left undetermined in this study.

Here we introduce Property Value (Vp). A Property has a meaning of < hyperglycemia, existence > or < hyperglycemia, true>. The value as “existence/nonexistence” or “true/false” should be independent of the degree value. However, because adding another form to the degree value would make the system of representation forms more complex, we deal with the degree value (e.g., mild/moderate/severe) as the specialization of the state of hyperglycemia; we treat these values of “existence/nonexistence” or “true/ false” in the same manner as the degree value. Consequently, we use Vp to represent both Vd and Vp.

The use of the value is e.g., a representation of the condition of “not being hyperglycemia,” < hyperglycemia, false>, in latent diabetes. For this reason, we consider that the true/false value is needed for computer processing.

In conclusion, our model provided the following interoperable representation forms:

(1) The < OAV > form as clinical test data

(2) The < OPV > form as abnormal states and

(3) The extended form < OSoAV > as clinical test data and < OSoPV > as abnormal states

Consequently, the OPV form is the same as the OAV form. Therefore, the OPV can be compliant with the unified representation (OAV triplet), which realizes the interoperability between the data and abnormal states.

### *Is-a* Hierarchy of abnormality ontology

Ontologically, the structural abnormality, dysfunction, pathological conditions, pathological processes, etc. are kinds of abnormal states. We propose abnormality ontology, which is a comprehensive ontology that covers all of the abovementioned concepts, and abnormal states are defined as the top level.

**Figure 1:**
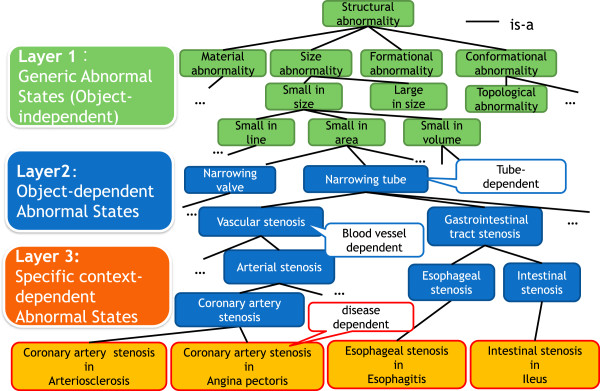
**Three-level ontological model of abnormal states.** This figure shows an example of the three levels of structural abnormality of our abnormality ontology. “Level 1” defines generic concepts, which are object-independent states, e.g., “small in area.” “Level 2” defines object-dependent abnormal states. States at the upper levels of Level 2 are dependent on generic structures, such as the “narrowing tube” and “narrowing valve,” which are common and are used in several domains. Note that concepts at the lower level of the tree are specialized into medicine-specific concepts such as vascular stenosis, arterial stenosis, and coronary artery stenosis. “Level 3” defines disease-dependent concepts. For example, “coronary artery stenosis in angina pectoris” is defined as a constituent of the disease “angina pectoris” at Layer 3.

Clinicians work with strongly domain-specific knowledge, which causes difficulties in finding common and generic knowledge across domains. A clear distinction between the basic/generic and specific concepts is required to be made. To this end, we propose the following three levels of abnormal states (Figure 1):

• Level 1: Generic abnormal states

• Level 2: Object-dependent abnormal states

• Level 3: Specific context-dependent abnormal states

### Level 1: generic abnormal states

Level 1 defines very basic (or generic) concepts, which do not depend on any structural entity, i.e., object-independent states. Examples include deformation, additional/missing anatomical structures, translocation, and dysfunction, which are commonly found in several objects, and can be usable in several domains besides medicine, such as machinery, materials, and aviation.

**Figure 2:**
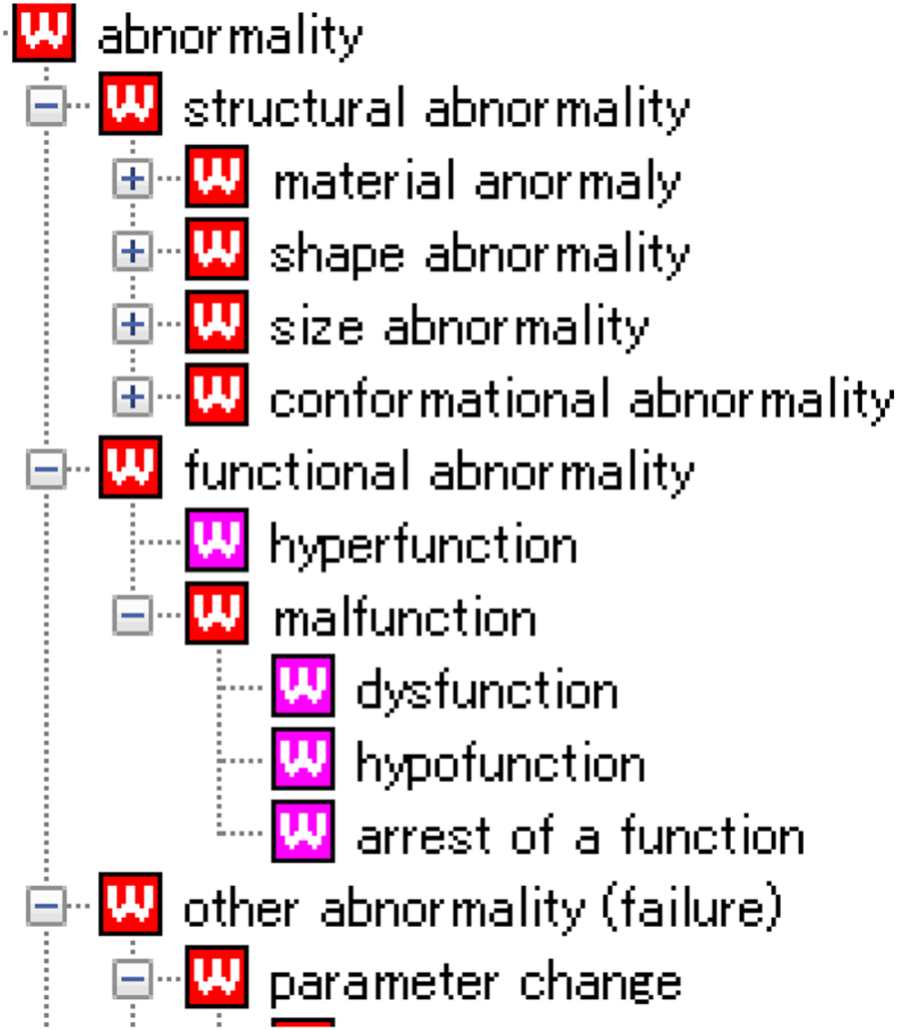
**Top-level categories related to abnormal states.** The top level categories of abnormal states are classified into three subclasses: “structural abnormality,” “functional abnormality,” and “other abnormality such as parametric/nonparametric change and so on”.

The top-level category of the generic abnormal states has three subclasses: “structural abnormality,” “functional abnormality,” and “other abnormality” (Figure 2). A structural abnormality is defined as an abnormal state associated with structure. It has subcategories of material abnormality (e.g., degeneration), shape abnormality (e.g., deformation), size abnormality, and conformational abnormality, such as topological abnormality (e.g., translocation), or structural defects (e.g., additional/missing structures) etc., while still retaining the identity of the structural body in question.

A functional abnormality is defined as an abnormal state that is related to an impaired function and is classified into hyperfunction and malfunction. Malfunction is subcategorized into dysfunction, function arrest, and hypofunction.

Other abnormal states include parametric abnormalities, which are classified into increased or decreased parameters, depending on whether or not the attribute has a higher or lower value than a threshold level. Examples included increased/decreased pressure or increased/decreased weight.

Our model has a recursive structure, in which the generic abnormal states at Level 1 are referred to by Level 2 object-dependent abnormal states.

### Level 2: Object-dependent abnormal states

Level 2 defines object-dependent abnormal states. The top level concepts at Level 2 are dependent on generic structures, such as “wall-type structure,” “tubular structure,” and “bursiform structure,” which are common and are used in several domains. Level 2 has been developed by identifying the target object and specializing generic abnormal states at Level 1 with consistency. For example, by specializing “small in area” at Level 1, “narrowing tube,” where the cross-sectional area has become narrow, is defined at Level 2 this is further specialized in the definitions “oil pipe narrowing” or “tracheal stenosis.”

In the lower layer of Level 2, abnormal states that are dependent on medical domain-specific objects, such as human anatomical structures, are defined and designed to represent concepts at all required granularities in the medical domain. Here in general, one problem arises in how fine the level of granularity needs to be supported in our ontology. In the case of “stenosis,” the term, “coronary artery stenosis” in a specific organ (the coronary artery) may be redundant. However, it is noteworthy that the abnormal states in one anatomical object can influence the adjacent objects, which causes other abnormal states. For example, although both are types of stenosis, coronary artery stenosis is different from rhinostenosis because the former causes myocardial ischemia and ischemic heart disease, whereas the latter causes sleep apnea. Therefore, there is a need for distinct abnormal states at specific organ levels.

From an ontological engineering point of view, our framework for modeling abnormal states is intended to capture the abnormal states from generic to specific levels, so as to provide abnormal states at the required granularity of specific organ/tissue/cell layers in the medical domain.

Here, the abnormal states of a specific object defined at Level 2 should be distinct from the disease-dependent concepts at Level 3. For example, hyperglycemia is defined in a context-independent manner at Level 2, and this is referred to in Level 3 concepts in various diseases, such as diabetes, metabolic syndrome, and lipodystrophy.

Currently, we are developing and enriching Level 2 concepts to link each Level 3 concept to the upper common level concept.

### Level 3: specific context-dependent abnormal states

Level 3 consists of context-dependent abnormal states, which refer to the Level 2 abnormal states, and are specialized into specific disease-dependent ones. For example, “rectal stenosis,” which is dependent on the rectum at Level 2, is defined as a constituent of Crohn’s disease at Level 3; this is also defined as a cause or an effect of other diseases, such as rectal cancer, Hirschsprung disease, or intestinal tuberculosis.

### Application work

#### Causal chains of disease

We have been developing a disease ontology, in which a disease is defined as a causal chain of abnormal states [9]. We divided the diseases into two major kinds: (1) ones where the etiological and pathological processes are well understood and (2) otherwise. Case (2) includes the so-called syndromes, typically represented in terms of the criteria for diagnosis. Diseases of type (1) is identified by its inherent etiological/pathological process(es). In this paper, we deal only with type (1) diseases. After careful examination of several diseases, we believe that every disease of type (1) should have a cue for identification. This means that we should be able to find the so-called main pathological/etiological condition(s), which theoretically characterize the disease to identify it. We know that diseases of type (2) necessarily employ criteria for diagnosis to identify the disease because of the lack of knowledge regarding etiological/pathological processes.

In addition, we believe that we need a formulation for organizing diseases in an *is-a* hierarchy in a disease model. According to the definition of a disease, this would consist of a causal chain(s), which consisted of nodes and links; a disease would be represented as a directed acyclic graph (DAG). We can introduce the *is-a* relation between diseases using the inclusion relationship between the causal chains as noted below.

##### *Is-a* relation between diseases

Disease A is a super class of disease B if all the causal chains at the class level of disease A are included in those of disease B. The inclusion of nodes (disorders) is judged by considering the *is-a* relation between the nodes into account as well as the sameness of the nodes.

##### Core causal chain of a disease

Causal chain(s) of a disease included in the chains of all its subclass diseases is called the core causal chain of a disease.

##### Derived causal chains of a disease

Causal chains of a disease defined as possible causal chains of abnormal states are called derived causal chains.

**Figure 3:**
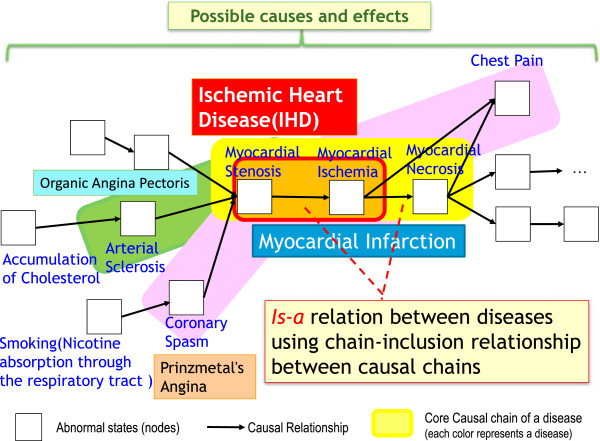
**Types of ischemic heart disease constituted of causal chains.** This figure shows a couple of causal chain-constituted ischemic heart disease. Each node shows the abnormal states, and each link indicates the causal relation between the abnormal states. A core causal chain of each disease is colored differently: ischemic heart disease is orange, and the subclasses of the ischemic heart disease, myocardial infarction are yellow. Prinzmetal angina is also a subclass of the ischemic heart disease consists of a pink core causal chain, and by an upstream extension smoking is added in the derived causal chain. Organic angina pectoris is green and the accumulation of cholesterol is added to the derived causal chain, which is a possible cause of arterial sclerosis.

For example, <myocardial stenosis → myocardial ischemia > is the core causal chain for ischemic heart disease and < myocardial stenosis → myocardial ischemia → myocardial necrosis > is the core causal chain for myocardial infarction. Here the core causal chain of Prinzmetal angina is defined as < coronary spasm → …>, and if there are some possible causes of spasm, e.g., smoking, it would be added to the upstream of causal chains as < smoking (nicotine absorption through the respiratory tract) → coronary spasm → … > as a derived causal chain (Figure 3).

Till date, clinicians have described the causal chains of diseases and abnormal states. We have been using these abnormal states to develop an *is-a* hierarchy of abnormalities. Abnormal states used in disease definitions in the ontology are defined as abnormal states at Level 3, where clinicians defined diseases in the respective medical departments. We collected all causal relationships from all disease concepts defined in the 13 medical departments and combined the causal chains, including the same abnormal states. As a result, the generic causal chains that contain all causal relationships, including approximately 21,000 abnormal states from 13 medical departments have been generated [14]. For example, we assume that a cardiovascular specialist in the division of cardiovascular medicine describes “coronary artery stenosis” and its causal chain as < coronary artery stenosis → myocardial ischemia → myocardial hypoxia > in ischemic cardiac disease. This can be linked with “coronary artery stenosis” in other diseases (e.g., hyperlipidemia) in other departments (metabolic medicine). As a result, a generic causal chain < accumulation of cholesterol → coronary artery stenosis → myocardial ischemia → myocardial hypoxia > of hyperlipidemia can be obtained as a possible causal relationship of abnormal states in the disease.

## Discussion and related work

### Discussion

We have introduced a unified form that represented an abnormal state as a time-indexed “Property,” and decomposed it into its “Attribute” and “Value”. Furthermore, we introduced the “Sub-Object,” which increases the flexibility with consistency. A property representation has several advantages. First, it easily captures the essentials of each disease because of its abstract nature. Second, it is relatively insusceptible to a small parameter modification. Third, it allows for the distinction between a definition of a disease and a diagnostic task that requires a quantitative representation.

Here, it should be noted that an abnormality can be explained as some bodily feature that is not part of the human life plan (unlike pregnancy) [15]; however, making a decision about whether or not a particular state is “abnormal” is not the job of ontologists but medical experts, who are required to make decisions on the basis of their medical knowledge. For example, answering a question about whether or not high HDL cholesterol level is an “abnormal state” would not be a task for ontologists but for medical experts; therefore, we do not discuss this issue in the present study.

We demonstrated that our model is interoperable between the quantitative and qualitative data found in several medical records, and the conceptual knowledge of the abnormal states in the definition of diseases.

In this study, we do not deal with the concrete value that is to be set for the threshold because thresholds may tend to change with time; for example, the cutoff value of the fasting plasma glucose (FPG) level was revised to 140 mg/dL in 1980 and to 126 in 1999 [16]. Therefore, we can freely change the threshold, and to do so is intrinsic. Nevertheless, even if the threshold changes, hyperglycemia will remain as < blood, glucose concentration, high >.

Diversity and heterogeneous representation problems of abnormal states are solved by a unified and consistent framework. However, in clinical DB, compound concepts are often found in clinical terms. For example, “blood pressure” is considered to be a compound concept, which consists of two elements “blood” (Object) and “pressure” (Attribute) joined for one meaning denoting an attribute. Other examples are “WBC count” and “blood glucose level”. Precisely speaking, these concepts should not be dealt with as an ontological matter but as a variation of data representation. Because medicine also requires an exchange of the real world data such as clinical test data between hospitals or institutions, in the next step, we will deal with them as a variation of data representation.

We have developed an ontology of abnormal states from generic to specific levels.

We have confirmed that abnormal states in the definition of 10 major diseases from three medical departments can be described with our description framework and succeeded in developing the *is-a* hierarchy from Level 1 to Level 3 in our preliminary work.

Till date, ontological engineers have defined Level 1 concepts together with a major portion of the higher levels of Level 2; clinicians have defined Level 3 concepts, including 21,000 abnormal states in 6,000 diseases in ontology. We plan to reformulate all the abnormal states at Level 3 in terms of our framework and complete the development of the middle concepts at Level 2 to link both the upper Level 1 and Level 3 abnormal states.

Some readers might think Level 3 is unnecessary and should be treated as the diagnostic instance level. However, by introducing Level 3 concepts, it will provide contextual information in the specific disease and contribute to the understanding of the background knowledge related to the underlying mechanisms of pathological process in the disease. Furthermore, Level 3 concepts are important for finding commonalities between the various diseases in terms of abnormal states. Therefore, we need to develop disease context-dependent levels as Level 3.

Our ontology is able to distinguish the common concepts from specific ones. Such an ontological approach contributes to finding commonalities not only across diseases in one division but also across departments. For example, in cardiovascular medicine, “coronary artery stenosis” in ischemic heart disease has a commonality with “pulmonary artery stenosis” in the tetralogy of Fallot in that they have the same upper abnormal state “arterial stenosis”. In addition, it has a commonality with “cerebrovascular stenosis” in brain infarction in cerebral surgery in that they both have the same upper abnormal state “vascular stenosis”. A further commonality can be found with “intestinal stenosis” in the ileus in gastroenterological medicine in that they have the same generic structure-dependent abnormal state “narrowing tube”. Therefore, finding commonalities across medical departments could offer a multidisciplinary perspective, allowing our method to be applied to a wide range of research.

In our application work, we have captured all 21,000 abnormal states across the 13 medical departments with both the *is-a* hierarchical structure of the abnormality ontology, and a causal chain as a relationship between different classes of abnormal states that are influenced by each other. This allows us to integrate fragmented knowledge of abnormal states, which might support the application of various kinds of medical knowledge, as follows.

(1) Conceptualization with little ambiguity

**Figure 4:**
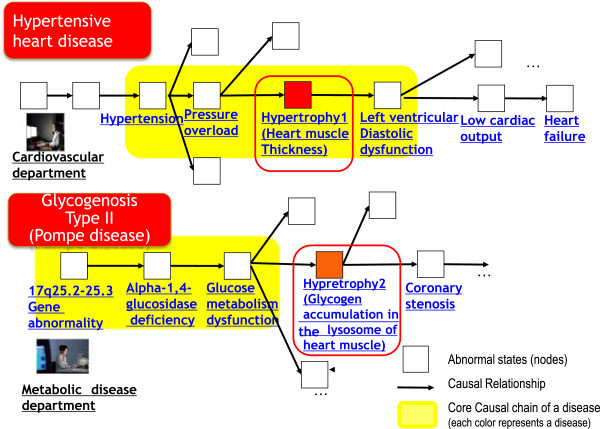
**Examples of hypertrophy constituted of causal chains.** This figure shows two different uses of cardiac hypertrophy. Each cardiac hypertrophy is red. One usage is a constituent of a causal chain of the hypertensive heart disease in the cardiovascular department (upper figure), and the other is a constituent of a glycogenesis type II disease (Pompe disease) in the metabolic disease department shown below (A core causal chain of each disease is yellow).

In the medical domain, there are quite a few ambiguous clinical terms with the same name but different meanings. One reason behind this is that clinicians use each term in the context of specific diseases in their own departments. For instance, the medical term “cardiac hypertrophy” is used in both a division of cardiovascular medicine and metabolic medicine. The definition of hypertensive heart disease in cardiovascular medicine indicates an increase in the thickness of the heart muscle, which results from a pressure overload caused by hypertension in the context of the heart. On the other hand, in glycogenosis II (Pompe diseases) in metabolic medicine, it implies a glycogen accumulation in the heart muscle, which is caused by metabolic dysfunction (Figure 4). Because our model can provide the appropriate upper levels of concepts and can give contextual information, it is possible to clarify their difference.

Thus, our model can reveal the context of the meanings that is usually hidden in the implicit background knowledge of clinicians, and will contribute to making a clear distinction between different types of concepts.

(2) Management of attributes by unified representation

If we allow clinicians to freely express the various attributes/abnormalities, it would lead to a lack of consistency and interoperability. Our model solves this problem by providing a unified representation model of attributes/abnormal states, as discussed in section Representation of abnormal states, in which the attributes and properties are differentiated; the properties are decomposed into < attribute, attribute value>, as well as the advanced representation for ratios and meta-attributes.

(3) Quantitative assessment of commonality

Traditionally, abnormal states have been dealt with in a manner specific to each disease in a particular medical division. Here, our model enables the capture of abnormal states common to several diseases, i.e., those that are at the first two levels and those that are disease-independent, which allows clinicians to overlook all abnormal states across medical departments.

As a result, we can quantify and assess the degree of commonality of abnormal states between different medical departments. In addition, it is possible to verify the commonality of generic concepts by abstracting, or to find disease-specific abnormal states with no commonality to any disease in other departments. For example, “esophagostenosis,” which is a subclass of “narrowing tube,” may demonstrate that it is specific to esophageal disease by showing no commonality with other diseases, whereas vascular stenosis can be confirmed as being more common by showing a higher rate of commonality across multiple diseases. Furthermore, our model may find commonalities of abnormal states that have always been treated as quite different abnormal states in different departments.

The clinicians’ treatment of the abnormal states in a manner specific to a disease and/or particular clinical division may have caused fragmentation of the same concept into different ones that are treated as differently. Because our approach finds commonalities in the organ-independent abnormal states, we can clean up and deal with abnormal states more simply.

Thus, our ontology will provide a clue to revealing the context embedded as background knowledge, which will allow us to compare abnormal states and evaluate their commonalities across medical departments.

### Related work

Upper ontologies such as BFO [1,2], DOLCE [3], and Galen [17] also deal with qualities and have contributed to dealing with the semantics of data. BFO formalizes < Entity, Property > (e.g., <rose, red>), whereas DOLCE uses < Entity, Attribute, Value > formalization (e.g., <rose, color, red>), and Galen adopts < Entity, Property, Value > formalization, (e.g., <rose, redness, high>). Phenotypic Quality (PATO) [6] is an ontology of phenotypic qualities, where the description was changed from < Entity, Attribute, Value > to < Entity, Property (Quality) > (e.g., <eye, red>) when they employed BFO. As explained in the Background section, the YAMATO ontology is an upper ontology in Japan [4], and offers interoperability among all of these descriptions, allowing us to handle all three kinds of descriptions in our representation model. Furthermore, PATO2YAMATO provides the mapping of ontology terms of PATO to YAMATO’s framework [5], and enables the interoperability of the quality framework between the different top-level ontologies such as BFO and DOLCE. In a preliminary study, the application has succeeded in making the connection between rat or mouse phenotype data, and related human abnormal states in the definition of diseases in this study [18]. Because our extended model enables the capture of commonalities of abnormal states across biological species, it may contribute to translational research linking mouse experimental data and clinical research.

Furthermore, on the basis of the ontological approach, if we make explicit the commonality and specificity of abnormal states among multi-species, it should support a comprehensive understanding of the basic common mechanism or principles underlying organisms, and would lead to scientific discoveries by acquiring biomedical knowledge through an interdisciplinary approach across species.

In the medical domain, medical ontologies and standard vocabularies, such as ICD-10 [19], SNOMED-CT [20], have been developed and extensively used in practice. However, they are largely based on legacy system terminologies, and thus have some ontological problems [21]. SNOMED-CT is a comprehensive terminology, which contains more than 311,000 clinical terms. However, it is not compliant with any formal upper level ontology. SNOMED-CT allows for multiple inheritance that causes a messy situation in the classification of entities, despite the fact that partitioning implies sibling classes are mutually disjoint, siblings at lower levels overlap each other, which results in complex taxonomic graphs and maintenance of the ontology difficult [20]. Furthermore, SNOMED-CT does not distinguish disorders from diseases. Not all disorders are diseases.

Our ontological proposal will help avoid such problems. On the basis of YAMATO, we systematically define abnormal states from the generic level (Level 1) to the specific anatomical structure-dependent level (Level 2). Furthermore, by specializing Level 2 concepts into a disease-context (disease-specific) level (Level 3), we can distinguish abnormal states from diseases.

In our future plan, our ontology will be translated into English and be mapped with SNOMED-CT clinical terms. The mapping will evaluate the standard terminologies in line with fundamental ontology engineering and provide useful information about causal relationships of abnormal states in the definition of each disease.

LOINC [8] provides the universal code names and clinical terms by decomposing them. However, because it focuses on the clinical observations, several of the abnormal states appearing in the definition of diseases are out of the scope for LOINC.

Although LOINC has < O (SO) A > like our model, it does not have Value (V). For example, a test for glucose tolerance about after 2 hours serum glucose for 100 g oral is represented by “GLUCOSE^2H POST 100 G GLUCOSE PO:MCNC:PT:SER/PLAS:QN”. The aim of LOINC is to standardize the vocabulary for the representation of clinical test data and is useful for interoperability among various data. However, our claim is not isolated to adopting the OAV form. In order to realize the interoperability between the clinical test data and abnormal states, a Quantitative Value (Vqt) is needed in the representation form. Our model can deal with quantitative data in the OAV form; therefore, we can transform it into the OP form of abnormal states. As a result, our model has an ability to maintain the interoperability between the clinical test data to abnormal states in diseases. Our model is not merely a theoretical contribution. Only reutilizing the existing resources cannot realize the interoperability between the various representation forms. We need more sophisticated organization of related representations including quantitative and qualitative data to exploit all of them in a consistent manner. To the best of our knowledge, our model is the first to make such an exploitation possible, which will contribute to medical practices.

Our model contributes to the systematization of abnormal states on the basis of ontological theory, and is able to distinguish between generic abnormal states, object-dependent ones, and disease-specific ones with unified representation. Moreover, the generic abnormal states are referred to the lower level of abnormal states by specializing them into the required granularity. In the future, we plan to examine mappings to other data sets of representations of clinical observations such as in LOINC or MEDIS [22] that have been opened to the public by The Medical Information System Development Center in Japan (MEDIS-DC) for interoperability. These mappings would provide a more comprehensive analysis of interoperability between the clinical observation data and the conceptual knowledge of abnormal states in the definition of diseases.

OGMS, which uses BFO as an upper-level ontology [23], and DO [24] are both medical ontologies. However, they do not have causal relationships between the abnormal states in one disease. Our strategy will contribute to providing a good resource for several medical researchers to analyze the causes of diseases from the viewpoint of the causal relationships of the abnormal states. Collaborative efforts in OBO Foundry have tried to coordinate various ontologies to support biomedical data integration [25]. In the next step, we plan to convert our abnormal state ontology into OBO format, and provide useful information about the causal relationships in diseases.

In our practical application work, we published some parts of the causal chain in disease ontology as Linked Open Data (Disease Chain LOD) on the basis of our RDF model [26]. It includes definitions of 2,103 diseases and 13,910 abnormal states in six major medical departments extracted from the disease ontology on May 11, 2013. In addition, we have developed the visualization system for disease chains called the Disease Chain LOD Viewer, which is available at http://lodc.med-ontology.jp/. Furthermore, a browsing system of disease chains with related information, which are obtained from other linked data or web service from external datasets (e.g., ICD 10, MeSH from DBPedia), is currently under development.

## Conclusions

We proposed a representation model of abnormal states designed in a unified manner. Our medical ontology project was started seven years ago. Since then, it has been refined and revised several times through discussion with both ontologists and clinicians. Till date, we have applied this model to approximately 21,000 abnormal states from approximately 6000 diseases.

We have demonstrated that our model has interoperability between quantitative and qualitative data and the conceptual knowledge of abnormal states in the definition of diseases. With this model, we have been developing an ontology of abnormal states from generic to specific levels. In the application we considered, we built disease chains consisting of causal relationships of abnormal states. By combining the disease chains and the ontology, we have captured all causal relations of the 21,000 abnormal states in the 6,000 diseases across 13 medical departments.

Although abnormal states have traditionally been considered to be specific to each disease in a particular medical department, our approach has found commonalities among abnormal states across medical departments.

## Methods

### Data sources for representation and ontology development of abnormal states

Medical doctors of The University of Tokyo Hospital described the disease ontology, and definitions of diseases were determined. Medical dictionaries [10,27] and textbooks [28,29] were used as references. Using them as resources, the definitions of the abnormal states in the definition of diseases were decided through repeated discussions by ontology engineers and medical experts. The Level 1 generic concepts were based on YAMATO [4]; the top level concepts at Level 2, i.e., generic object-dependent concepts, have been generated by ontologists. In our Japanese medical ontology project, anatomical entities have been defined [30], and their anatomical structure-dependent abnormal states have been defined by the medical experts. Level 3 disease-specific abnormal states were collected and generated by clinicians in 13 special fields.

### Ontology editing tool

**Figure 5:**
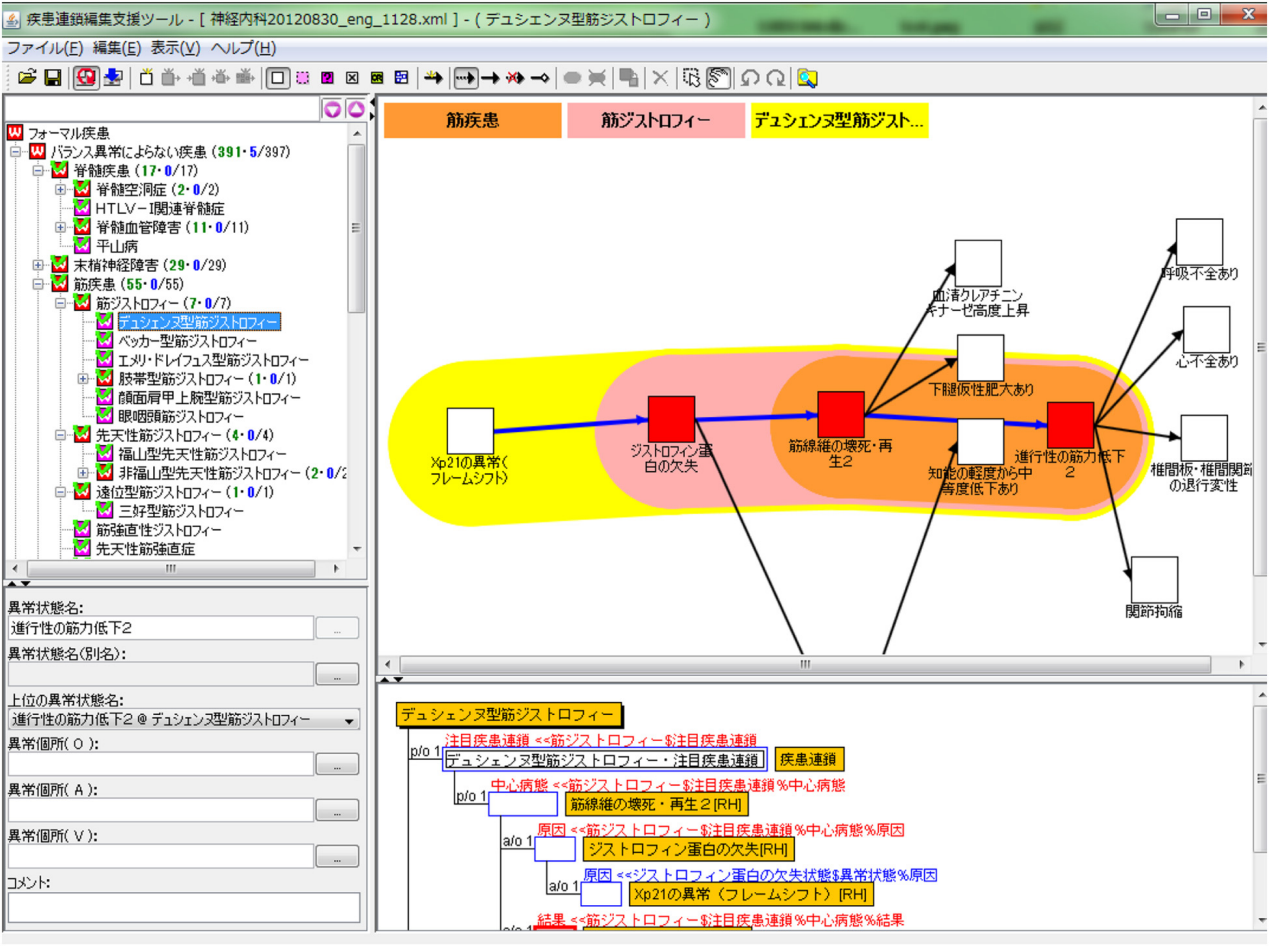
**Computational representation of abnormal states from generic to specific level.** This figure shows the specialization of abnormal states from “small in area” to “ischemic heart disease specific coronary artery stenosis” using HOZO.

Ontology editing was performed using HOZO [31]. An example of specialization of an abnormal state from a generic level to a specific level is shown in Figure 5. The generic abnormal state “Small in area” at Level 1 is defined by < area (Attribute), small (Qualitative Value)>. Furthermore, at the top level concepts of Level 2, by identifying the target object as “tubular structure,” and specializing it into < cross-sectional area, small>,” we can define “narrowing tube,” where the cross-sectional area of tube became narrow. Lower concepts at Level 2 are specialized to represent abnormal states specific to human anatomical structures. For example, “vascular stenosis,” which is dependent on “blood vessels,” is further specialized into “coronary artery stenosis,” which is dependent on “coronary artery.” Furthermore, “coronary artery stenosis” at Level 2 is specialized into a disease-dependent one at Level 3; for example, ischemic heart disease dependent. In ischemic heart disease, coronary artery stenosis causes “decreased blood flow,” which results in “myocardial ischemia”.

### Visual input/editing tool

**Figure 6:**
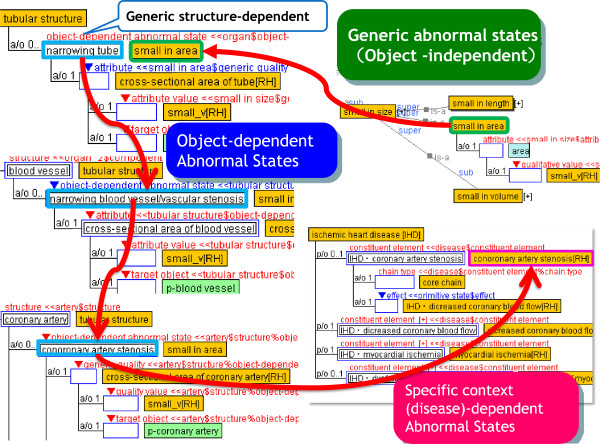
**A visual editing tool for causal chains to define disease concepts.** This is a screenshot of our visual editing tool for editing the definition of disease concepts. It visualizes the causal chains defined in a selected disease as a directed graph like that in Figure 3.

We developed a visual editing tool, so that clinicians can easily input and edit the definition of disease concepts. Figure 6 shows its user interface. It enables us to visualize the causal chains defined in a selected disease as a directed graph. In the graph, nodes represent the abnormal states and links represent causal relationships between them. When users edit the graph, it automatically translates into ontology according to the HOZO’s format. The ontology can be exported in the OWL format by the export functions of HOZO.

Although it has been implemented as a client application using HOZO’s ontology API, we have published the part of disease ontology as Linked Open Data with SPARQL endpoint to get their causal chains [32]. The Disease Chain LOD is available at the URL http://lodc.med-ontology.jp/.

## Endnotes

^a^Ontologically, a state corresponds to a time-indexed property. However, in the definition of diseases, time-indexed abnormal states are rarely used (e.g., ischemia in the definition of ischemic heart disease)” because the time-dependence of properties of diseases is out of the scope of our project.

^b^In this paper, although we mainly deal with a simple parameter, additional parameters can be used when necessary. For example, in the definition of neonatal anemia, we associate an additional attribute “age” and the value “neonatal” with it.

## Competing interests

The authors declare that they have no competing interests.

## Authors’ contributions

YY, designed the representation frame work, developed the ontology of abnormal states, and drafted the manuscript. KK designed the representation frame work, developed the ontology of abnormal states, and helped to draft the manuscript. TI and KO have led our medical ontology project and developed the disease ontology. RM defined most of the theory and helped to draft the manuscript. All authors read and approved the final manuscript.
